# Low prevalence of active cytomegalovirus infection in a cardiovascular intensive care unit

**DOI:** 10.1186/2052-0492-2-12

**Published:** 2014-02-18

**Authors:** Haruhiko Ishioka, Masamitsu Sanui, Yusuke Tsutsumi, Fumitaka Yanase, Junji Shiotsuka

**Affiliations:** Department of Anesthesiology and Critical Care Medicine, Jichi Medical University Saitama Medical Center, Amanumacho, Omiya-ku, Saitama, 330-8503 Japan; Division of Molecular Epidemiology, Jikei University School of Medicine, Nishishimbashi, Minato-ku, Tokyo, 105-8461 Japan

**Keywords:** Cytomegalovirus, Antigen, Incidence, Epidemiology, Cardiovascular surgery, Intensive care unit

## Abstract

Active cytomegalovirus infection is not uncommon in critically ill non-immunosuppressed patients. We conducted a preliminary observational study to determine the prevalence of active cytomegalovirus infection in cardiovascular surgical patients. One hundred patients admitted to the intensive care unit following cardiovascular surgery were enrolled between January 2010 and May 2010. Four patients (4%) were positive for serum pp65 antigens, though cytomegalovirus-positive serology (immunoglobulin G, IgG) was found in 98 patients (98%) including those four patients. Active cardiac diseases and their operative procedures including cardiopulmonary bypass may not be significant risk factors for active cytomegalovirus infection unless systemic derangements are also present.

## Findings

Active cytomegalovirus (CMV) infection, diagnosed by one of the following examinations for CMV, either pp65 antigen, polymerase chain reaction, or viral culture, is not as uncommon in critically ill non-immunosuppressed patients, as previously thought [1]. Recent studies have also shown that CMV infection may play a role in the progression of cardiovascular diseases [2, 3]. Although there are several reports about an association of CMV virus infection with active cardiovascular conditions [4, 5], the existence of this association is still uncertain. We therefore conducted a preliminary observational study to determine the prevalence of active CMV infection in cardiovascular surgical patients.

### Methods and results

One hundred patients admitted to our intensive care unit (ICU) following cardiovascular surgery between January 2010 and May 2010 were enrolled. Serum pp65 antigens for CMV at ICU admission, day 7, and day 14 were evaluated. A serum pp65 antigen-positive was defined as ≥1 cell per 100,000 leukocytes. CMV serology (immunoglobulin G, IgG) tests were also performed on admission. This study was approved by the ethical committee of Jichi Medical University. Written informed consent was obtained from patients. Statistical analysis was performed with Stata V13. The differences in characteristics were tested using Fisher’s exact test for categorical variables and the Wilcoxon test for continuous variables.

Four of 100 patients (4.0%) were positive for serum pp65 antigens, while CMV-positive serology (IgG) was found in 98 patients (98.0%) including those four patients. Thus, the incidence of reactivation was 4.1% among IgG-positive patients. The demographic details of all IgG-positive patients are outlined in Table 1. Characteristics of antigen-positive patients included: patient 1 had a history of rheumatic arthritis on long-term corticosteroids, who underwent elective aortic valve replacement with perioperative steroid supplementation; patient 2 had a diagnosis of native-valve endocarditis due to methicillin-resistant *Staphylococcus aureus* (MRSA), which required emergency aortic valve replacement; patient 3 underwent Y graft replacement, complicated with postoperative acute kidney injury (AKI) due to left gluteal muscle ischemia and rhabdomyolysis; Patient 4 had a history of receiving adjuvant chemotherapy with orally active fluoropyrimidine against residual gastric cancer, who underwent off-pump coronary artery bypass grafting before radical gastrectomy. Patients 2 and 4 had positive pp65 antigens at ICU admission, while patient 1 became positive on day 7, and patient 3 became positive on day 14. Quantitative values of pp65 antigenemia were 1 positive per 100,000 leukocytes in all of four patients. No clinically significant changes in positive cell counts were detected up to day 14 in either patient. No clinical signs or symptoms of end-organ disease were seen during the postoperative course among these four patients, and no specific treatment was given after positive pp65 antigen testing. All antigen-positive patients except patient 3 received intraoperative blood transfusion. The median number of acute physiology and chronic health evaluation (APACHE) II scores on admission in those four patients were similar to the antigen-negative patients (18.5 [interquartile range (IQR), 8.8–20.8] vs 16.0 [IQR, 12.0–19.0]). One of the antigen-negative patients died during the hospital stay.

**Table 1:** **Demographics of CMV IgG-positive patients**

	Negative CMV infection (***n*** = 94)	Positive CMV infection (***n*** = 4)	***P***value^a^
Age, mean ± SD	66.7 ± 10.2	68.5 ± 7.1	0.727
Female gender, *n* (%)	22 (23)	2 (50)	0.251
Comorbidities			
Hypertension, *n* (%)	61 (65)	3 (75)	1.000
Diabetes mellitus, *n* (%)	25 (27)	0	0.570
Chronic kidney disease (stage 5), *n* (%)	10 (11)	1 (25)	0.384
Malignancy, *n* (%)	1 (1)	1 (25)	0.080
Preoperative severe infection, *n* (%)	0	1 (25)	0.041
Recent corticosteroid use (<1 mo), *n* (%)	0	1 (25)	0.041
Duration of operation (min), median (IQR)	328 (275–393)	315 (259–415)	0.993
Duration of cardiopulmonary bypass (min), median (IQR)	130 (0–172)	53 (0–118)	0.194
APACHE II scores, median (IQR)	16.0 (12.0–19.0)	18.5 (11.5–20.5)	0.559
Length of ICU stay (day), median (IQR)	3.0 (3.0–5.0)	5.0 (3.5–8.0)	0.179
Length of postoperative stay (day), median (IQR)	15.0 (13.3–21.0)	19.0 (18.0–43.0)	0.090
In-hospital mortality, *n* (%)	1 (1)	0	1.000

APACHE, acute physiology and chronic health evaluation; IQR, interquartile range. ^a^Fisher’s exact test for categorical variables, Wilcoxon test for continuous variables.

### Discussion

According to these preliminary results, a few cardiovascular surgical patients have active CMV infection despite the high prevalence of CMV IgG antibody in the perioperative period. CMV reactivation is often induced by immunosuppressive process such as AIDS or immunosuppressive therapies for solid organ and hematopoietic stem cell transplantation, but in critically ill non-immunosuppressed patients, immunological derangement associated with critical illness, so-called immunoparalysis, can reactivate CMV [1, 6]. Each patient with active CMV infection in this study had each risk factor including steroid use, severe infection, postoperative AKI, or chemotherapy. The presence of these known risk factors could influence the immunological status of the patients. Chemotherapy could induce CMV reactivation [7]. Steroid exposure in the ICU could be a potential reactivation trigger as well [8]. Timing of the first positive results for pp65 antigen in those patients might be related to the duration of their underlying diseases and the severity, presumably because CMV requires a substantial time period to proceed from the latent phase to active infection. Intraoperative transfusion could have induced their positive result, especially for the two patients who were antigen-positive at the time of ICU admission, although filtered leukocyte-reduced blood products, which may reduce the risk of CMV transmission [9], have been used in our hospital. The limitations of this preliminary study are the small number of patients and a relatively short length of the study period. Optimal monitoring should have been more than 21 days to detect later reactivations according to previous results [5].

The low prevalence of active CMV infection in this study population could suggest relatively low patient severity, reflected by a median APACHE II score of 16. According to a subgroup analysis of the previous systematic review [1], a low prevalence of active CMV infection was confirmed in non-immunosuppressed intensive care unit patients with relatively low disease severity. Active cardiac diseases and their operative procedures including cardiopulmonary bypass may not be risk factors for active CMV infection unless systemic derangements are also present.

## Abbreviations

AIDS: Acquired immune deficiency syndrome
AKI: Acute kidney injury
APACHE: Acute physiology and chronic health evaluation
CMV: Cytomegalovirus
ICU: Intensive care unit
IQR: Interquartile range
MRSA: Methicillin-resistant *Staphylococcus aureus.*

## References

[1] Kalil AC, Florescu DF (2009). Prevalence and mortality associated with cytomegalovirus infection in nonimmunosuppressed patients in the intensive care unit. Crit Care Med.

[2] Smieja M, Gnarpe J, Lonn E, Gnarpe H, Olsson G, Yi Q, Dzavik V, McQueen M, Yusuf S, Heart Outcomes Prevention Evaluation (HOPE) Study Investigators (2003). Multiple infections and subsequent cardiovascular events in the Heart Outcomes Prevention Evaluation (HOPE) study. Circulation.

[3] Crumpacker CS, Zhang JL, Mandell GL, Bennett JE, Dolin R (2010). Cytomegalovirus. Principles and Practice of Infectious Diseases.

[4] Prösch S, Wendt CE, Reinke P, Priemer C, Oppert M, Krüger DH, Volk HD, Döcke WD (2000). A novel link between stress and human cytomegalovirus (HCMV) infection: sympathetic hyperactivity stimulates HCMV activation. Virology.

[5] Limaye AP, Kirby KA, Rubenfeld GD, Leisenring WM, Bulger EM, Neff MJ, Gibran NS, Huang ML, Santo Hayes TK, Corey L, Boeckh M (2008). Cytomegalovirus reactivation in critically Ill immunocompetent patients. JAMA.

[6] Hotchkiss RS, Karl IE (2003). The pathophysiology and treatment of sepsis. N Engl J Med.

[7] Kuo CP, Wu CL, Ho HT, Chen CG, Liu SI, Lu YT (2008). Detection of cytomegalovirus reactivation in cancer patients receiving chemotherapy. Clin Microbiol Infect.

[8] Cook CH, Trgovcich J (2011). Cytomegalovirus reactivation in critically ill immunocompetent hosts: a decade of progress and remaining challenges. Antiviral Res.

[9] Bowden RA, Slichter SJ, Sayers M, Weisdorf D, Cays M, Schoch G, Banaji M, Haake R, Welk K, Fisher L, McCullough J, Miller W (1995). A comparison of filtered leukocyte-reduced and cytomegalovirus (CMV) seronegative blood products for the prevention of transfusion-associated CMV infection after marrow transplant. Blood.

